# Application of a decision tree model in the early identification of severe patients with severe fever with thrombocytopenia syndrome

**DOI:** 10.1371/journal.pone.0255033

**Published:** 2021-07-30

**Authors:** Bohao Wang, Zhiquan He, Zhijie Yi, Chun Yuan, Wenshuai Suo, Shujun Pei, Yi Li, Hongxia Ma, Haifeng Wang, Bianli Xu, Wanshen Guo, Xueyong Huang

**Affiliations:** 1 College of Public Health, Zhengzhou University, Zhengzhou, China; 2 Henan Province Center for Disease Control and Prevention, Zhengzhou, China; 3 Joint Logistics Support Force NO.990 Hospital, Xinyang, China; 4 Henan Key Laboratory of Pathogenic Microorganisms, Zhengzhou, China; Tufts University Cummings School of Veterinary Medicine, UNITED STATES

## Abstract

**Background:**

Severe fever with thrombocytopenia syndrome (SFTS) is a serious infectious disease with a fatality of up to 30%. To identify the severity of SFTS precisely and quickly is important in clinical practice.

**Methods:**

From June to July 2020, 71 patients admitted to the Infectious Department of Joint Logistics Support Force No. 990 Hospital were enrolled in this study. The most frequently observed symptoms and laboratory parameters on admission were collected by investigating patients’ electronic records. Decision trees were built to identify the severity of SFTS. Accuracy and Youden’s index were calculated to evaluate the identification capacity of the models.

**Results:**

Clinical characteristics, including body temperature (p = 0.011), the size of the lymphadenectasis (p = 0.021), and cough (p = 0.017), and neurologic symptoms, including lassitude (p<0.001), limb tremor (p<0.001), hypersomnia (p = 0.009), coma (p = 0.018) and dysphoria (p = 0.008), were significantly different between the mild and severe groups. As for laboratory parameters, PLT (p = 0.006), AST (p<0.001), LDH (p<0.001), and CK (p = 0.003) were significantly different between the mild and severe groups of SFTS patients. A decision tree based on laboratory parameters and one based on demographic and clinical characteristics were built. Comparing with the decision tree based on demographic and clinical characteristics, the decision tree based on laboratory parameters had a stronger prediction capacity because of its higher accuracy and Youden’s index.

**Conclusion:**

Decision trees can be applied to predict the severity of SFTS.

## Introduction

Severe fever with thrombocytopenia syndrome (SFTS) is a tick-born infectious disease caused by a novel virus called SFTS virus (SFTSV) [1]. SFTS was first reported in China in 2006, and other Asian countries, such as Japan and Korea, reported it later [2–4]. SFTSV is a novel member of the Phlebovirus genus of the Phenuiviridae family, which can be transmitted by several transmissions, including tick bites and person-to-person transmission through blood or other body fluids [5–9]. The average fatality rate of SFTS is up to 30% [10], which makes SFTS a serious health threat, and the World Health Organization lists SFTSV as a priority pathogen requiring urgent attention.

According to previous research, there is no specific antiviral therapy for SFTSV infection, and the most essential parts of case management are symptomatic treatment and supportive therapy [11]. The timely referral of serious SFTS patients to the intensive care unit (ICU) has been associated with an increased survival rate [12]. It is important for physicians to recognize patients who are experiencing severe situations as early as possible due to the high fatality of SFTS. An early and accurate identification model for the severity of SFTS could not only illustrate parameters that influence the evolution of SFTS but also help clinicians make better decisions and improve the efficiency of the treatment.

Decision trees are one of the most effective methods for data mining, as extracting meaningful information from measured data represents a plausible solution for massive data learning tasks [13]. Furthermore, decision trees have the advantages of a nonparametric setup, the tolerance of heterogeneous data, and immunity to noise [14]. This study aims to develop a parsimonious model in the form of a decision tree for classifying the severity of SFTS using the most frequently observed symptoms and laboratory parameters on admission.

## Methods

### Patients

From June to July 2020, a total of 86 suspected patients admitted to the Infectious Department of the Joint Logistics Support Force No. 990 Hospital, and 71 of them were identified as laboratory-confirmed patients by PCR assay. The real-time PCR assay using PCR Diagnostic Kit for SFTSV RNA (BGI-GBI, China) was performed as previously described [15, 16].Of the 71 cases, all patients were farmers from Xinyang or an adjacent administrative region, 40 (56.3%) patients were males and 31 (43.7%) patients were females. The mean age of the patients was (62.58±11.89) years.

### Inclusion criteria

According to the “Guideline for SFTS prevention and control (2010)” issued by the National Health and Family Planning Commission, SFTS cases were diagnosed by the following criteria: (1) epidemiological characteristics (e.g., history of tick bites, working in mountainous areas or teahouses, or direct contact with the blood of a confirmed patient during the two weeks prior to symptom onset); (2) clinical presentation (e.g., fever (>38°C), headache, muscle aches, nausea, vomiting, diarrhea, skin bruising, bleeding, multiple-organ damage); (3) laboratory findings (e.g., decrease in leukocyte count and thrombocytopenia); and (4) The exclusion criterion was if the patient was positive assessed through PCR assay of acute phase blood samples for other diseases such as hemorrhagic fever with renal syndrome (HFRS), dengue fever, and thrombocytopenic. A patient who met all of the above criteria was diagnosed with SFTS [16].

Patients were divided into two groups: mild and severe. Severe SFTS patients were defined as any patient who either required admission to an ICU or met at least one of the following criteria: a) acute lung injury (ALI) or acute respiratory distress syndrome (ARDS); b) heart failure; c) acute renal failure; d) encephalitis; e) shock; and f) disseminated intravascular coagulation (DIC) or death [17].

### Data collection

All patients’ electronic records were investigated, and serum samples were collected at admission. All samples were transported frozen to the pathogen laboratory of Henan Center for Disease Control and Prevention (Henan CDC). Patient demographic information, including age, sex, exposure history, history of tick bite, clinical characteristics (body temperature, cough, nausea, muscular aches, fatigue), and routine laboratory parameter results, was collected by investigating the patient’s electronic record.

### Ethics approval and consent to participate

The research was approved by the Ethics Committee of Henan Center for Disease Control and Prevention. All participants gave written informed consent for the use of their samples in research. All data analyzed were anonymized.

### Decision trees

Decision trees have proven to be a valuable tool for extracting meaningful information from measured data and represent a plausible solution for massive data learning tasks [18]. There are three classic decision tree algorithms: ID3, C4.5, and CART. C4.5 and CART can handle both continuous variables and discrete variables, and they are not sensitive to incomplete data, whereas ID3 can only handle discrete variables [19]. CART generates binary trees, and C4.5 generates multiple branches. In this study, CART was employed to construct a prediction model. The details of the CART algorithm are as follows.

Suppose that there are *C* categories of data in sample dataset *S*. The Gini index formula is as follows:

$$Gini(S)=1-\sum_{i=1}^{c}P_{i}^{2}$$

where *S* represents the training data set, *C* represents the data class number and *P*_*i*_ represents the ratio of the sample number in class *i* to all samples. Technically, suppose the current node corresponds to the training data set *S*, and characteristic root *v* divides *S* into *k* disjoint subsets *S*_*1*_, *S*_*2*_, *S*_*3*_, *…*, *S*_*K*_, that is,

$$S=S_{1}\cup S_{2}\cup S_{3}\cup \ldots \cup S_{k}$$

The information gain G(S, v) is as follows:

$$G(S,v)=Gini(S)-\sum_{i=1}^{k}\frac{\left|S_{i}\right|}{\left|S\right|}Gini(S_{i})$$

Since Gini(*S_i_*)≥0, we have *G*(*S, v*)≤Gini(*S*). According to the definition of information gain, more information gain means stronger classification capacity.

### Statistical analysis

SPSS 21.0 and R 4.0.2 were applied to perform statistical analysis. Categorical variables are summarized as frequencies and proportions. Continuous variables with a normal distribution are described as mean and standard deviation (SD), whereas those with an abnormal distribution are described as median and interquartile range (IQR). The unpaired t test or Mann–Whitney U test was employed to test the differences in continuous variables between the mild and severe cases. Comparisons of the clinical parameters between the two groups were carried out by the Pearson χ^2^ (when sample size was over 40 and theoretical frequencies were over 5)or Fisher exact test(when sample size was smaller than 40 or theoretical frequencies were smaller than 1) in tables. The predictive value of models was evaluated by indexes including accuracy and Youden’s index. A P value < 0.05 was considered to be statistically significant.

## Results

### Patients’ demographic and clinical characteristics

**Fig 1:**
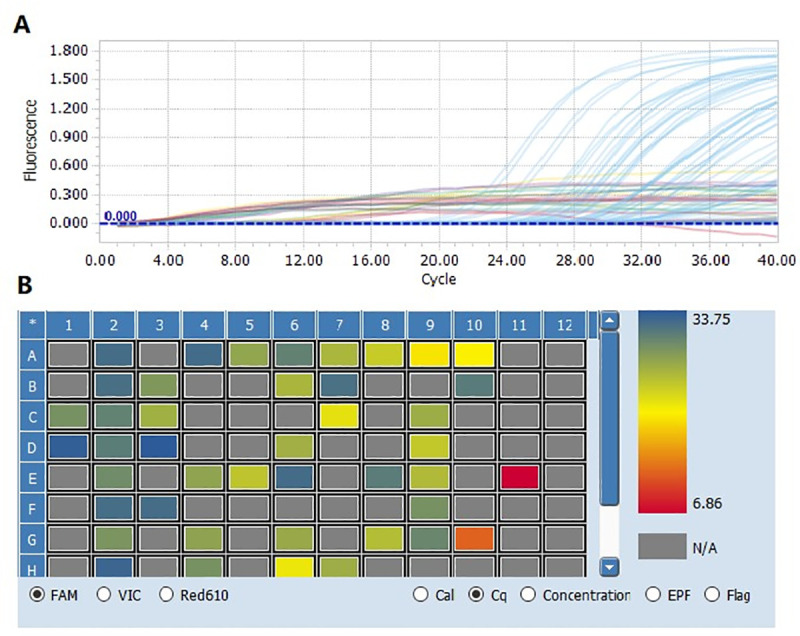
FTSV RNA assessed through RT-PCR assay of partial suspected patients.

Through PCR assay, a total of 71 SFTS patients were enrolled (Fig 1), including 30 mild patients and 41 severe patients. There was no difference in sex distribution between the two groups (χ^2^ = 1.975, p = 0.160). There was no significant difference in age between the two groups (t = -1.643, p = 0.105).

The major clinical adverse events included fever (n = 52, 73.24%), cough (n = 20, 28.2%), fatigue (n = 69, 97.2%), nausea (n = 56, 78.9%), muscular aches (n = 54, 76.1%) and lymphadenectasis (n = 41, 57.7%). Gastrointestinal symptoms included poor appetite (n = 68, 95.8%), vomiting (n = 41, 57.7), stomachache (n = 28, 39.4%), and diarrhea (n = 37, 52.1%). Neurologic symptoms included lassitude (n = 62, 87.3%), limb tremor (n = 25, 35.2%), hypersomnia (n = 12, 16.9%), discordance of consciousness and coma (n = 7, 9.9%), and dysphoria (n = 9, 12.7%).

The differences in body temperature and the size of the lymphadenectasis between the mild and severe groups were statistically significant (F = 7.832, p = 0.011; F = 6.581, p = 0.021, respectively). There was a significant difference in the proportion of patients with cough between the two groups (χ^2^ = 5.651, p = 0.017). All gastrointestinal symptoms were comparable in mild and severe patients; conversely, most of the differences in neurologic symptoms between the two groups were statistically significant, including lassitude (χ^2^ = 14.085, p<0.001), limb tremor (χ^2^ = 18.555, p<0.001), hypersomnia (χ^2^ = 6.810, p = 0.009), discordance of consciousness and coma (χ^2^ = 5.682, p = 0.017), and dysphoria (χ^2^ = 7.541, p = 0.006). Details of the patients’ demographic and clinical characteristics are shown in Table 1.

**Table 1:** Demographic and clinical characteristics of SFTS patients.

Characteristics	Mild n = 30	Severe n = 41	p value
Demographic features			
Age (y)	59.9±13.9	64.54±9.9	0.105
Male/female	14/16	26/15	0.160
Clinical characteristics			
Body temperature (°C)	38 (36–39)	38 (36–39)	0.011
Size of lymphadenectasis (cm^2^)	0 (0–2)	0 (0–6)	0.021
Cough	4 (13.3%)	16 (39.0%)	0.017
Fatigue	29 (96.7%)	40 (97.6%)	0.822
Nausea	24 (80.0%)	32 (78.0%)	0.842
Muscular aches	22 (73.3%)	32 (78.0%)	0.646
Gastrointestinal symptoms			
Poor appetite	29 (96.7%)	39 (95.1%)	0.749
Vomit	18 (60.0%)	23 (56.1%)	0.742
Stomachache	10 (33.3%)	18 (43.9%)	0.368
Diarrhea	12 (40.0%)	25 (61.0%)	0.081
Neurologic symptoms			
Lassitude	21 (70.0%)	41 (100.0%)	<0.001
Limb tremor	2 (6.7%)	23 (56.1%)	<0.001
Hypersomnia	1 (3.3%)	11 (26.8%)	0.009
Coma	0 (0)	7 (17.1%)	0.018
Febrile	0 (0)	9 (22.0%)	0.008

* Age was described as (mean±SD); body temperature and size of lymphadenectasis were described as median (IQR).

### Comparison of laboratory testing results between mild and severe cases

Of all laboratory testing resluts, platelet count (PLT), lactate dehydrogenase (LDH), aspartate aminotransferase (AST), and creatine kinase (CK) in severe patients were significantly different from those in mild patients. PLT was lower in severe patients than mild patients, whereas AST, LDH and CK were dramatically higher. Other laboratory features (e.g., white blood cells (WBCs), lymphocytes (LYMs), neutrophils (NEUs), alanine aminotransferase (ALT), gamma-glutamyl transpeptidase (GGT), and creatinine (Cr)) were comparable between the two groups. Details of the comparison of laboratory features are shown in Table 2.

**Table 2:** Laboratory parameters in mild and severe patients.

Parameters	Mild n = 30	Severe n = 41	p value
PLT (10^9^/L)	68.5 (36.5, 89.0)	38.0 (27.5, 52.0)	0.006
WBC (10^9^/L)	4.0 (3.0, 6.0)	3.0 (2.0, 5.0)	0.301
LYM%	29.77±15.29	29.83±16.08	0.987
NEU%	62.90±17.84	59.78±20.30	0.503
AST (U/L)	97.5 (50.0, 160.3)	205.0 (128.5, 313.5)	<0.001
ALT (U/L)	48.5 (35.0, 96.3)	85.0 (56.5, 139.0)	0.114
GGT (U/L)	27.5 (18.8, 55.8)	44.0 (29.5, 70.0)	0.097
LDH (U/L)	511.57±250.02	867.29±437.15	<0.001
Cr (μmol/L)	66.70±15.72	75.0±54.58	0.422
CK (U/L)	133.0 (69.0, 261.25)	450.0 (204.0, 1325.0)	0.003

* LYM%, NEU%, LDH and Cr were described as (mean±SD); PLT, WBC, AST, ALT, GGT and CK were described as median (IQR).

### Decision tree analysis

**Fig 2:**
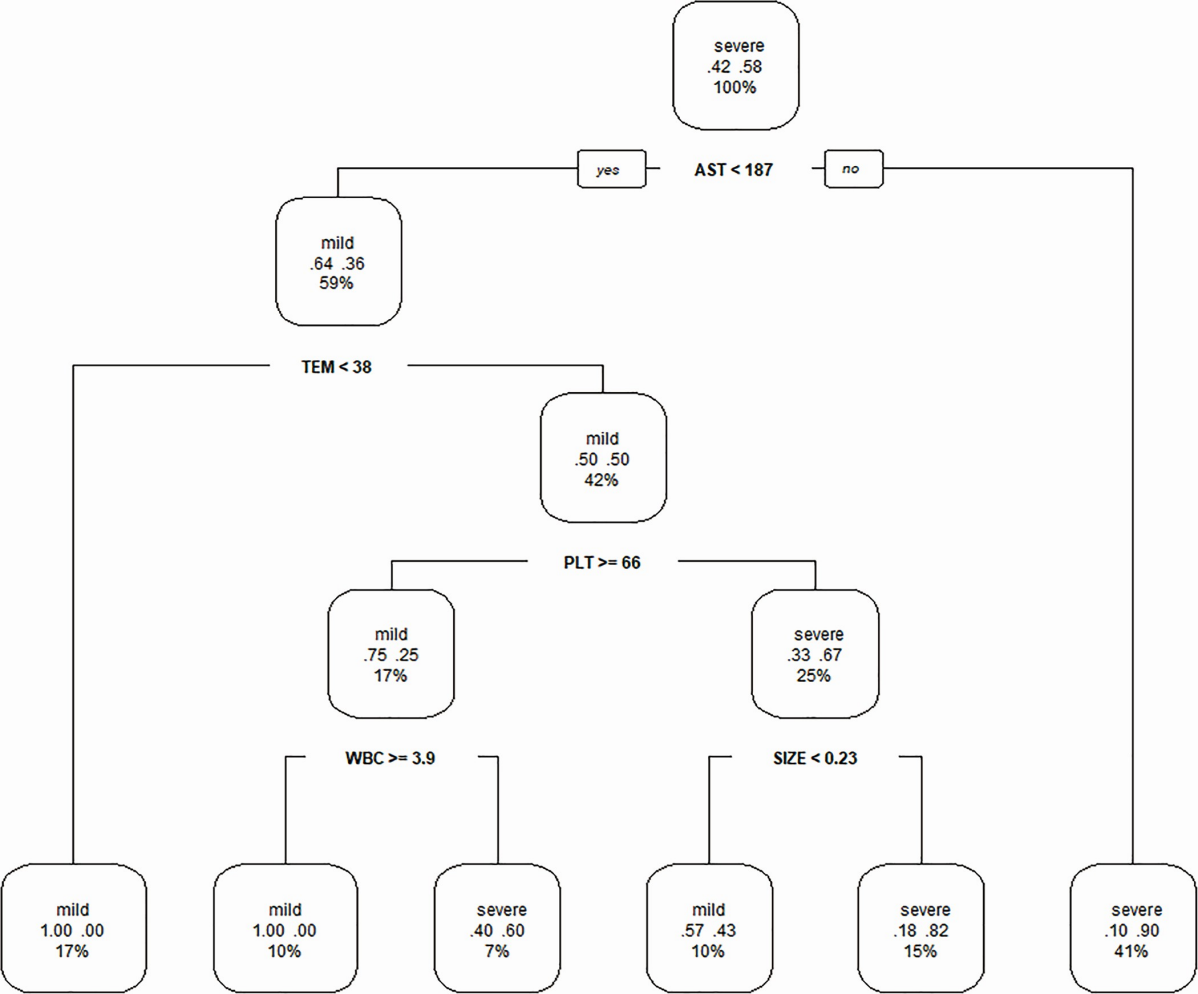
Decision tree based on laboratory parameters of SFTS patients. TEM = body temperature; SIZE = size of lymphadenectasis.

The first decision tree, based on laboratory parameters generated by the optimized CART algorithm, is shown in Fig 2. Body temperature and lymphadenectasis size were included with the laboratory parameters because they were continuous variables. The decision tree had five depth and six leaf nodes. AST, body temperature, PLT, WBC and size of lymphadenectasis were included as discriminating factors in the decision tree for predicting the severity of SFTS after five splits: split (a): all 71 enrolled patients were divided based on AST<187 U/L (n = 42) or AST≥187 U/L (n = 29); split (b): patients were divided based on body temperature <38°C (n = 12) or body temperature ≥38°C (n = 30); split (c): patients were divided based on PLT≥66×10^9^/L(n = 12) or PLT<66×10^9^/L(n = 18); split (d): patients were divided based on WBC≥3.9×10^9^/L (n = 7) or WBC<3.9×10^9^/L (n = 5); split (e): patients were classified into severe if the size of the lymphadenectasis was ≥0.23 cm^2^ (n = 11) and mild if the size of the lymphadenectasis was <0.23 cm^2^).

**Fig 3:**
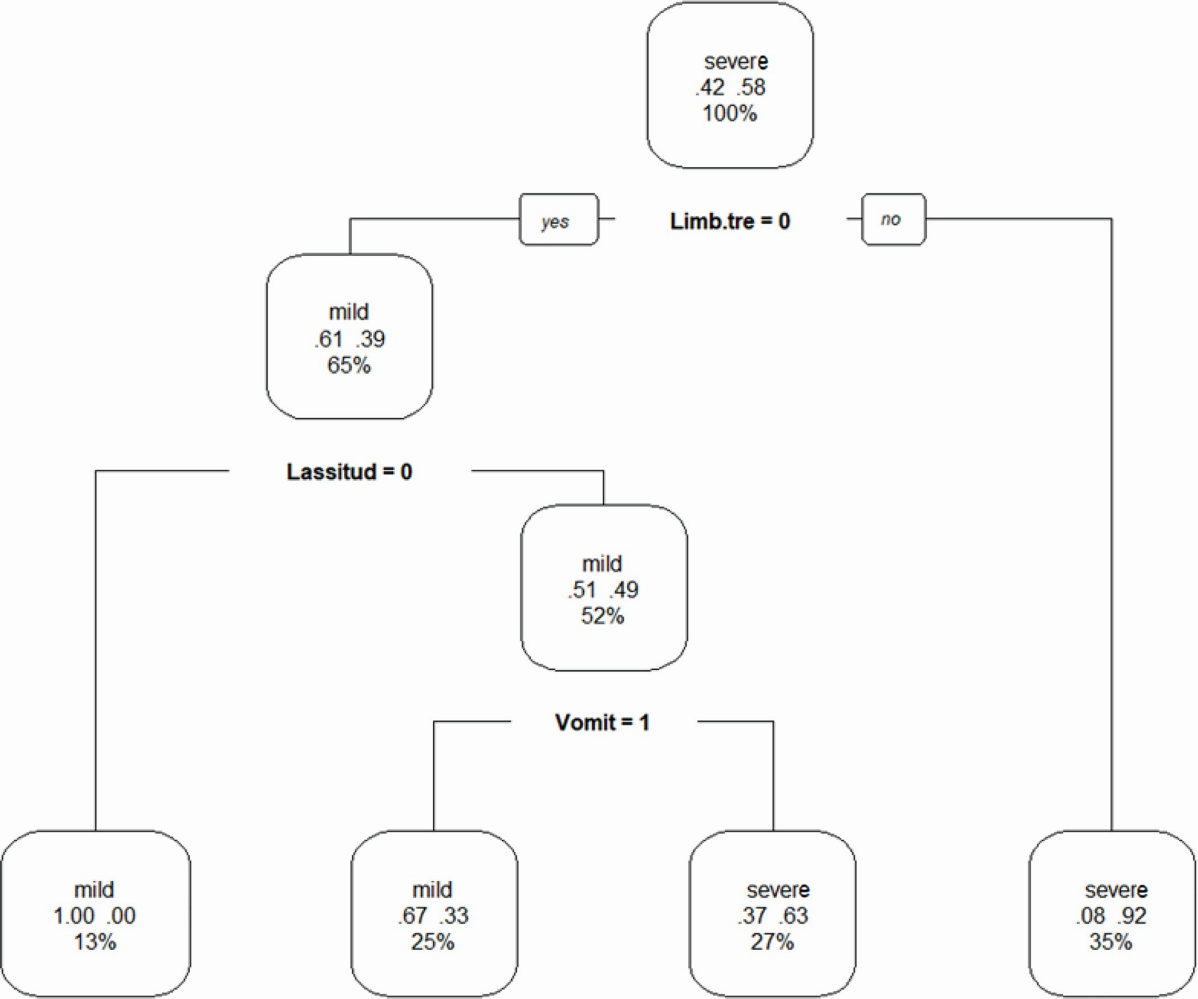
Decision tree based on demographic and clinical characteristics of SFTS patients. Limb.tre = limb tremor; 1 = ‘YES’, 0 = ‘NO’.

The second decision tree was generated based on the demographic and clinical characteristics of SFTS patients. Limb tremor, lassitude and vomiting were included as discriminating factors in the decision tree and formed four groups. Split (a): if patients had limb tremor, then they were severe; split (b): if patients had lassitude, then they were mild; split (c): if patients had vomiting, then they were severe. Details of the second decision tree are shown in Fig 3 below.

### Validation of the models

Cross-validation was performed to select the optimal model, whose test set has the lowest error rate. Cross-validation can make maximum use of the acquired information, which is to divide the training set *S* into *k* non-overlapping subsets *S*_*1*_, *S*_*2*_, *…*, *S*_*k*_. In the k-repeat validation, each of the subset is selected as the test set in sequence, and the remaining data is used as the training set to train the model.

The performance of the decision trees was evaluated by confusion matrices based on true positives (TP: number of patients with severe SFTS who were correctly predicted), true negatives (TN: number of patients with mild SFTS who were correctly predicted), false positives (FP: number of patients with mild SFTS who were wrongly predicted to have a severe condition), and false negatives (FN: number of patients with severe SFTS who were wrongly predicted to have a mild condition). The sensitivity, specificity, accuracy and Youden’s index were calculated based on the abovementioned parameters. These criteria were calculated as follows:

$$Accuracy=\frac{TP+TN}{TP+TN+FP+FN}$$

$$Sensitivity=\frac{TP}{TP+FN}$$

$$Specificity=\frac{TN}{TN+FP}$$

$$Youden^{\prime }s\;index=Sensitivity-(1-Specificity)=(Sensitivity+Sensitivity)-1$$

The confusion matrices of the two decision trees are summarized in Table 3. The sensitivity, specificity, accuracy and Youden’s index of the two decision trees are displayed in Table 4. The decision tree based on the laboratory parameters of SFTS patients achieved a sensitivity of 92.7% and a specificity of 70.0%. The sensitivity and specificity of the decision tree based on demographic and clinical characteristics of the SFTS patients were 82.9% and 73.3%, respectively. According to Table 4, the first decision tree had a higher accuracy, which meant that the decision tree based on laboratory parameters had a more efficient classification strategy.

**Table 3:** Confusion matrices of the two decision trees.

		Decision tree 1		Decision tree 2	
		Severe	Mild	Severe	Mild
Predicted	Positive	38	9	34	8
	Negative	3	21	7	22

Decision tree 1 was based on laboratory parameters of SFTS patients; decision tree 2 was based on demographic and clinical characteristics of SFTS patients.

**Table 4:** Sensitivity, specificity, accuracy and Youden’s index of the two decision trees.

	Sensitivity%	Specificity%	Accuracy%	Youden’s index
Decision tree 1	92.7	70.0	83.1	0.63
Decision tree 2	82.9	73.3	78.9	0.56

Decision tree 1 was based on laboratory parameters of SFTS patients; decision tree 2 was based on demographic and clinical characteristics of SFTS patients.

## Discussion

The main clinical manifestations of SFTS are fever, fatigue, and muscular aches [20], and some patients also develop nausea at the initial stage of the disease, making it similar to many other viral infections. Like other hemorrhagic fevers, SFTS can also cause gastrointestinal symptoms, leukopenia, thrombocytopenia, elevated tissue enzymes, and hematuria proteinuria [16], which makes it difficult to diagnose and treat patients. SFTS patients are mostly middle-aged and elderly farmers. In severe cases, neurological symptoms and bleeding symptoms may occur, and patients may even die due to multiple-organ failure [21]. The number of fatal cases has increased annually in China, although national intervention programs that promote public awareness, set up sentinel hospitals and improve clinicians’ skills have been established [22]. Therefore, accurate prediction of the prognosis may help clinicians perform intervention measures in advance, control the disease progression and improve the prognosis.

In this study, demographic and clinical characteristics and laboratory parameters were compared between mild and severe SFTS patients. Univariate analysis showed that the body temperature and size of lymphadenectasis in severe patients were higher than those in mild patients. Cough, lassitude, limb tremor, hypersomnia, coma and dysphoria were risk factors for severity in SFTS patients. Although none of the gastrointestinal symptoms were significantly different between the two groups, in the decision tree based on demographic and clinical characteristics, vomiting was included as a discriminating factor. In the decision tree based on laboratory parameters, WBC was included as a discriminating factor, though it had no significant difference between mild and severe patients. This phenomenon demonstrates one of the usages of decision trees: variable selection [23]. Decision tree methods can be used to select the most relevant input variables that can be used to formulate clinical hypotheses and inform subsequent research, similar to stepwise variable selection in regression analysis.

**Fig 4:**
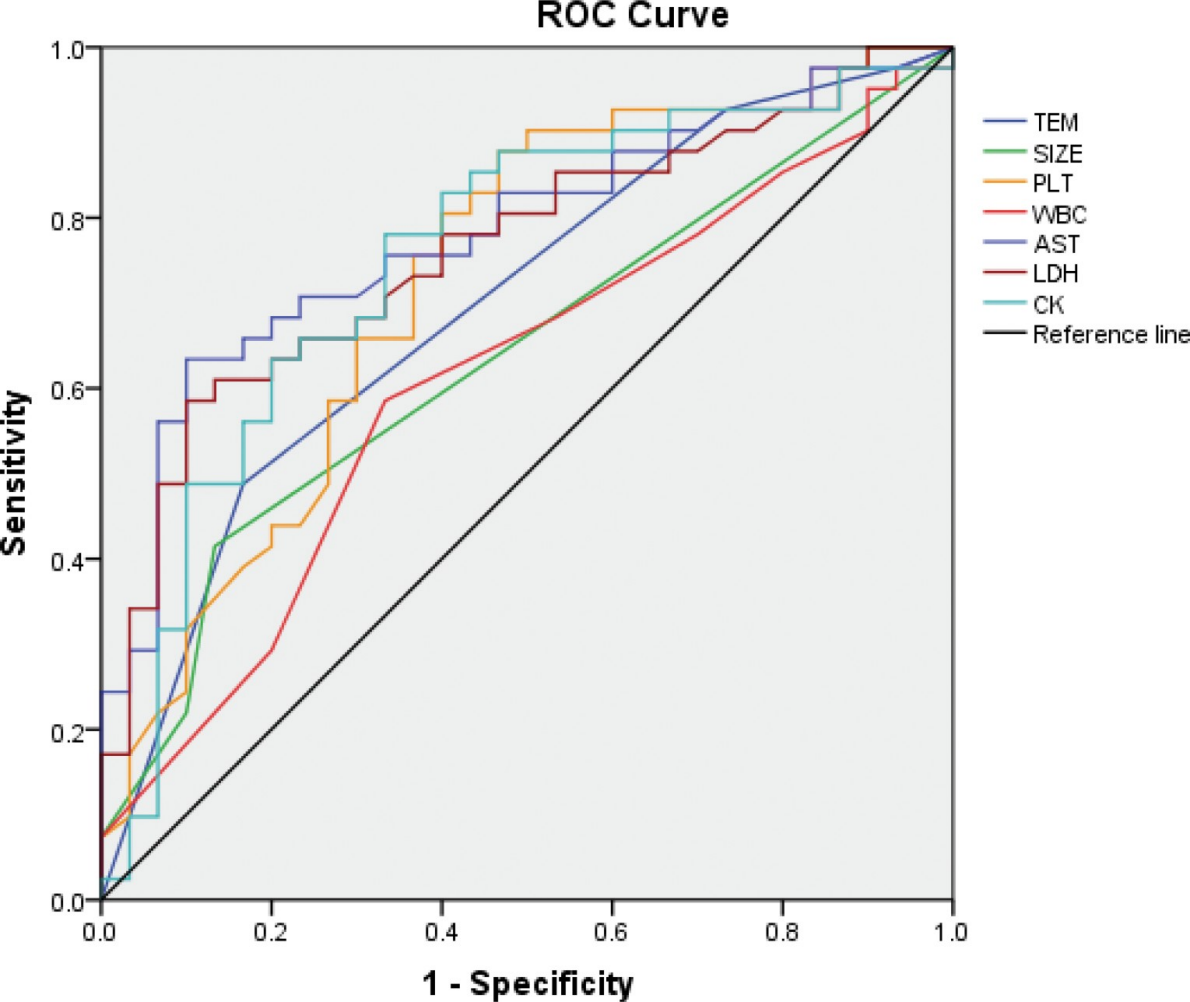
ROC curves for the prediction of the severity of SFTS.

To our best knowledge, there are four prediction models for SFTS patients, and most of them predict death [11, 12, 22, 24]. According to previous studies, ALT, AST, CK, LDH and Cr levels are critical risk factors for fatal SFTS patients [11, 22, 24–26]. Consistent with previous studies, differences in PLT, AST, LDH and CK between mild and severe patients in this study were significant. The size of lymphadenectasis is also a critical factor in diagnosing SFTS from other hemorrhagic fevers in clinical practice. The performance of the prediction based on the independent factors is shown in Fig 4 and Table 5. Though these are significant factors for predicting the severity of SFTS, decision trees had a stronger classification capacity due to their higher Youden’s index.

**Table 5:** Cutoff values and AUCs of different parameters for the prediction of severity of SFTS, with their sensitivity and specificity.

Parameters	Cutoff value	AUCs(95%CI)	Sensitivity%	Specificity%	Youden’s index
TEM (°C)	38.5	0.698(0.574,0.821)	48.8	83.3	0.321
SIZE (cm^2^)	0.5	0.638(0.509,0.767)	41.5	86.7	0.282
PLT (10^9^/L)	65.5	0.728(0.607,0.849)	87.8	53.3	0.411
WBC (10^9^/L)	3.42	0.610(0.478,0.743)	58.5	33.3	0.252
AST (U/L)	186.5	0.782(0.675,0.889)	63.4	90.0	0.534
LDH (U/L)	765.5	0.763(0.652,0.874)	58.5	90.0	0.485
CK (U/L)	202.5	0.754(0.637,0.872)	78.0	66.7	0.447

In conclusion, decision trees can be applied to predict the severity of SFTS. Body temperature, size of the lymphadenectasis, PLT, AST, LDH and CK are classification factors whose prediction capacities are lower than those of decision trees. The classification strategy of the decision tree based on laboratory parameters was more efficient than the classification strategy of the decision tree based on clinical and demographic characteristics.

## Supporting information

S1 Data (XLSX)
